# Rhizospheric *Actinomycetes* Revealed Antifungal and Plant-Growth-Promoting Activities under Controlled Environment

**DOI:** 10.3390/plants11141872

**Published:** 2022-07-18

**Authors:** Hazem S. Elshafie, Ippolito Camele

**Affiliations:** School of Agricultural, Forestry, Food and Environmental Sciences, University of Basilicata, Viale dell’Ateneo Lucano 10, 85100 Potenza, Italy; hazem.elshafie@unibas.it

**Keywords:** biocontrol, phytopathogens, bioactive substances, microbial biostimulants, antagonistic activity, *Actinobacteria*

## Abstract

*Actinomycetes* has large habitats and can be isolated from terrestrial soil, rhizospheres of plant roots, and marine sediments. *Actinomycetes* produce several bioactive secondary metabolites with antibacterial, antifungal, and antiviral properties. In this study, some *Actinomycetes* strains were isolated from the rhizosphere zone of four different plant species: rosemary, acacia, strawberry, and olive. The antagonistic activity of all isolates was screened in vitro against *Escherichia coli* and *Bacillus megaterium*. Isolates with the strongest bioactivity potential were selected and molecularly identified as *Streptomyces* sp., *Streptomyces atratus*, and *Arthrobacter humicola*. The growth-promoting activity of the selected *Actinomycetes* isolates was in vivo evaluated on tomato plants and for disease control against *Sclerotinia sclerotiorum*. The results demonstrated that all bacterized plants with the studied *Actinomycetes* isolates were able to promote the tomato seedlings’ growth, showing high values of ecophysiological parameters. In particular, the bacterized seedlings with *Streptomyces* sp. and *A. humicola* showed low disease incidence of *S. sclerotiorum* infection (0.3% and 0.2%, respectively), whereas those bacterized with *S. atratus* showed a moderate disease incidence (7.6%) compared with the positive control (36.8%). In addition, the ability of the studied *Actinomycetes* to produce extracellular hydrolytic enzymes was verified. The results showed that *A. humicola* was able to produce chitinase, glucanase, and protease, whereas *Streptomyces* sp. and *S. atratus* produced amylase and pectinase at high and moderate levels, respectively. This study highlights the value of the studied isolates in providing bioactive metabolites and extracellular hydrolytic enzymes, indicating their potential application as fungal-biocontrol agents.

## 1. Introduction

Recently, new agrochemical drugs have been registered in agriculture field, but they can have different negative effects on plants, the environment, and humans. Furthermore, several phytopathogenic microorganisms have become resistant to some agrochemicals, which requires the development of new antimicrobial agents to avoid this serious phenomenon [1,2]. Currently, many scientists all over the world are trying to discover new natural drugs of plant or microbial origin [3,4,5,6,7]. Many plant and microorganisms produce different bioactive secondary metabolites that can potentially be used in the agro-pharmaceutical industry as efficient alternatives for several chemical pesticides [3,8,9,10].

The soil is a rich matrix of living microorganisms and is a valuable resource of biological control agents [11,12,13]. The rhizosphere, which is made up of aggregates containing accumulated organic matter, is a repository of microbial activity in the soil. The rhizosphere has great importance because it can support large populations of active microorganisms [14]. Furthermore, soil microorganisms provide an excellent source for important bioactive products [15]. There is growing interest in using bacteria for medicinal and agricultural purposes due to their ability to produce a wide range of biologically active substances with antibiotic, fungicidal, herbicidal, hydrolytic enzymatic, antitumor, antivirals, and immune-suppressant activities [16,17,18]. Recently, pathogen resistance has necessitated the discovery of new antimicrobial agents effective against bacteria and fungi. There is strong interest in screening new microorganisms from different habitats for antimicrobial activity in order to discover new and promising antibiotics in the treatment against multi-drug resistant pathogens (MDRPs).

*Actinomycetes*, a type of unicellular Gram-positive bacteria, are widely distributed in nature from different habitats and are well-known and important producers of several bioactive secondary metabolites, antibiotics, and growth-promoting factors [19]. *Actinomycetes* are very similar to fungi, though they form hyphae much smaller than fungi [19,20]. The phylum *Actinobacteria* is considered one of the important groups of *Actinomycetes* [21,22]. Girão et al. [23] reported that many thousands of bioactive substances have been identified from *Actinobacteria*, especially those from terrestrial sources. The produced bioactive metabolites from *Actinomycetes*, especially those from terrestrial sources, represent about the 45% of known microbial bioactive metabolites [23,24]. In addition, Girão et al. [23] studied the antimicrobial activity of the organic extracts from some *Actinobacteria* isolated from *Laminaria ochroleucahe* and concluded that several isolates were able to inhibit the growth of *Candida albicans* and *Staphylococcus aureus*. *Streptomyces*, among the *Actinobacteria*, is considered an important genus able to produce the majority of the identified bioactive compounds, as reported by Berdy [25].

The isolation and biochemical characterization of *Actinomycetes* may allow finding new bioactive substances for pharmaceutical and agricultural purposes. The main objectives of the current study were to: (i) isolate and identify new strains of *Actinomycetes* from different soil habitats; (ii) evaluate the in vitro antagonistic effect of the tested isolates against some common phytopathogens; and (iii) evaluate the in vivo growth-promoting effect of the most bioactive isolates and their antifungal activity against *Sclerotinia sclerotiorum* on tomato seedlings.

## 2. Results

### 2.1. Isolation and Preliminary Screening

The isolation from the soil samples allowed obtaining ten pure *Actinomycetes* isolates (Table 1). All isolates were preliminarily evaluated for their antagonistic activity against the two target microorganisms (*Escherichia coli* and *Bacillus megaterium*). The isolates AC1 and RS3 showed the highest biological activity against both tested microorganisms, whereas FG1 showed moderate activity against both tested microorganisms (Table 1). The OL2 isolate showed the highest activity against *E. coli* and the most promising activity against *B. megaterium* (Table 1). Based on the obtained results, the isolates AC1, RS3, and OL2 were selected for molecular identification and further biological assays.

### 2.2. Molecular Identification

The amplification with the primers Y1/Y2 produced amplicons with molecular weight of about 434 bp. No amplicons were observed in the negative control. The amplified DNA were sequenced (BMR Genomics, Padova, Italy), and the obtained sequences were compared with those available in GenBank nucleotide archive using Basic Local Alignment Search Tool software (BLAST) (RKV, MD, USA). The results of sequences analysis of AC1, RS3, and OL2 showed high similarity percentages to the sequences of *Streptomyces* sp., *Streptomyces atratus*, and *Arthrobacter humicola*, respectively, present in GenBank with the following accession numbers: ON241810, ON241816, and ON241806, respectively.

### 2.3. Extracellular Hydrolytic Enzymes

The results showed that all studied isolates were able to produce some extracellular hydrolytic enzymes (Table 2). In particular, the highest significant hydrolytic activity of chitinase (chitin azure), glucanase, and protease was observed in the case of *A. humicola*, where the diameter of the hydrolysis zones was 31.5, 36.0, and 21.5 mm, respectively. On the other hand, *Streptomyces* sp. and *S. atratus* showed the highest significant activity of amylase with a diameter of hydrolysis area of 37.5 and 42.0 mm, respectively, whereas the same two isolates showed moderate activity for pectinase with a diameter of hydrolysis area of 14.0 and 10.5 mm, respectively. However, *S. atratus* and *A. humicola* did not show either glucanase or pectinase activity, respectively. None of the three tested isolates showed hydrolytic activity for chitinase (chitin from crab shells) and polygalacturanase.

### 2.4. In Vivo Growth Promoting and Disease Control

#### 2.4.1. Eco-Physiological Parameters

The results revealed that all studied *Actinomycetes* isolates were able to stimulate the growth of bacterized tomato seedlings, which showed higher values of eco-physiological parameters in comparison with the negative control (non-bacterized plants), as represented in Table 3. In particular, seedlings inoculated with *Streptomyces* sp. and *A. humicola* showed the highest significant values (*p* < 0.05) of number of leaves, shoot length, shoot fresh weight, and shoot dry weight. The eco-physiological parameters of bacterized tomato seedlings artificially infected with *S. sclerotiorum* are reported in Table 4. In particular, seedlings inoculated with *Streptomyces* sp. and *A. humicola* demonstrated high values (*p* < 0.05) of number of leaves, twigs, shoot fresh weight, and shoot dry weight. However, *S. atratus* showed a moderate growth-promoting effect on tomato seedlings, especially in terms of the number of twigs, shoot length, and total shoot dry weight.

#### 2.4.2. Disease Control

**Figure 1:**
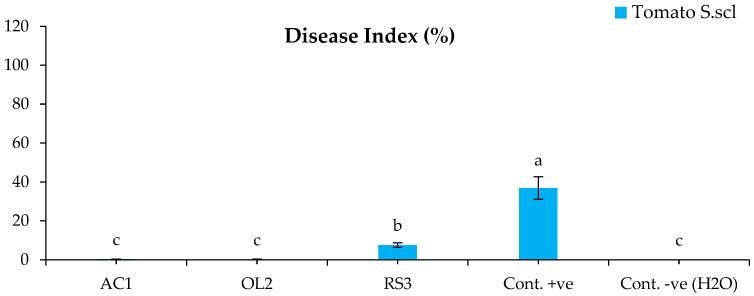
Disease index of tomato inoculated with *S. sclerotiorum*. Bars with different letters indicate mean values significantly different at *p* < 0.05 according to *Tukey’s* B test. Data are expressed as mean of 3 replicates ± SDs. DI (%) = [Σ (Scale × No. of SL)/(HS × TL)] × 100 (Equation (1)). AC1, OL2, and RS3 are *Streptomyces* sp., *Arthrobacter humicola*, and *Streptomyces atratus*, respectively.

**Figure 2:**
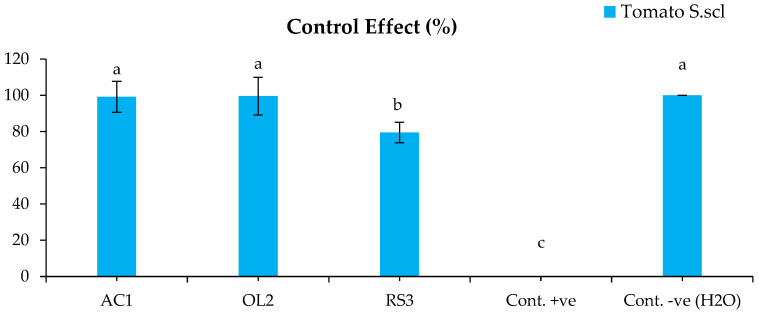
Control effect of the tomato inoculated with *S. sclerotiorum*. Bars with different letters indicate mean values significantly different at *p* < 0.05 according to Tukey’s test. Data are expressed as mean of 3 replicates ± SDs. CE (%) = 100 × (DI-P-DI-B)/DI-P (Equation (2)).

The bacterized plants with *Streptomyces* sp. and *A. humicola* did not show any symptoms on their leaves and roots after the infection with *S. sclerotiorum*. The disease indexes of the plants bacterized with *Streptomyces* sp. and *A. humicola* were 0.3% and 0.2% (Figure 1), whereas the control effects were 99.2% and 99.5%, respectively (Figure 2). The seedlings bacterized with *S. atratus* showed a moderate disease index of 7.6% (Figure 1) and a control effect of 79.5% (Figure 2). Regarding the positive control (plants inoculated only with *S. sclerotiorum*), the results showed the development of leaf yellowing and chlorosis at 20 DAI, where the leaf chlorotic zone of infected tomato plants became necrotic. Moreover, complete leaf wilting and root necrosis was also observed at 35 DAI. In particular, a significantly higher symptomatic leaves percentage (*p* < 0.05) was observed in the positive control, where the disease index was 36.8% compared with the negative control and bacterized plants with *Actinomycetes* isolates (Figure 1). *S. sclerotiorum* was always re-isolated from the inoculated plants.

## 3. Discussion

The obtained results proved that the studied *Actinomycetes* isolates were able to promote the growth of tomato plants by improving the eco-physiological characteristics and also are promising for the biocontrol of *S. sclerotiorum* on tomato seedlings. In particular, the biological activity and growth-promoting effect of the studied *Actinomycetes* strains may be due to their ability to produce some bioactive metabolites, such as growth hormones, which enhance the tomato seedlings’ growth [26,27,28,29]. The application of microbial plant stimulants is considered an important strategy for sustainable agriculture systems for enhancing plant growth and increasing production, especially under abiotic stress [30].

Sousa and Olivares [31] concluded that plant-growth-promoting Streptomyces (PGPS) was able to biostimulate plant growth by direct and indirect pathways such as phytohormones production, phosphate solubilization, and alleviation of various abiotic stresses. In particular, endophytic *Actinobacteria* can biostimulate the secretion of plant growth hormones such as indole acetic acid (IAA), as reported by Manulis et al. [32] and Dochhil et al. [33].

The treatments with *Streptomyces* sp. and *Arthrobacter humicola* showed high reduction in disease symptoms on tomato seedlings against the tested pathogenic fungi. Furthermore, the bacterization treatments induced a significant disease protection of tomato seedlings compared with non-bacterized plants against fungal infection with *S. sclerotiorum*. These results are in agreement with those of several researchers who reported that many soil-borne *Actinomycetes* are able to reduce the growth of some pathogenic fungi such as *Colletotrichum gloeosporioides*, *C. capsici*, and *Fusarium solani* f. sp. *pisi* [34,35,36,37].

The production of hydrolytic enzymes can also play an important role in controlling phytopathogenic fungi [38,39]. The cell wall lytic enzymes glucanase and protease can contribute to the degradation of fungal cell wall (skeletal) components through embedment in its protein matrix [40,41]. In addition, Ordentlich et al. [40] reported that chitinase and other lytic enzymes produced by *Serratia marcescens* were able to control the pathogenic fungus *Sclerotium rolfsii*, causing the release of *β*-glucanase, which can increase the chitinolytic activity in hyphal degradation [42].

Chaudhary et al. [43] studied the antagonistic activity of some *Actinomycetes* strains isolated from different niche habitats of Sheopur (India) and observed that some strains were highly active against *Bacillus cereus*, *Enterococcus faecalis*, *Shigella dysenteriae, Streptococcus pyogenes*, *Staphylococcus saprophyticus*, *S. epidermidis*, methicillin-resistant *Staphylococcus*, and *S. xylosus*. The same authors also reported that all studied isolates were able to inhibit the extracellular growth of tested microorganisms, whereas they were not able to inhibit intracellular growth of mycelium. The latter phenomena may be due to the production of some bioactive secondary metabolites that may not reach to the intracellular cells of the tested bacteria and hence were not able to denature their cell walls [43].

In a recent study conducted by Odumosu et al. [42], it was reported that some species of *Streptomyces* sp. showed promising antibacterial activity against some food and human pathogens such as *S. aureus*, *E. coli*, *Klebsiella pneumonia*, and *Salmonella typhi*. The same authors also chemically analyzed the secondary metabolites produced by the studied species using GC-MS and verified their antibiotic properties may be used in novel antimicrobials. The antifungal activity of *Streptomyces* strains may also be due to their ability to produce some bioactive secondary metabolites such as isoikarugamycin, a novel polycyclic tetramic acid macrolactam produced by *Streptomyces zhaozhouensis* active against *C. albicans*, as reported by Lacret et al. [44].

## 4. Materials and Methods

### 4.1. Soil Sampling and Isolation

For isolation of *Actinomycetes*, 200 g subsamples were collected from the rhizosphere zone of four different plant species: rosemary (3 samples), acacia (3 samples), strawberry (2 samples), and olive (2 samples) from Potenza (Basilicata region, southern Italy). Each soil sample was air-dried on the benches for one week and sieved through a 250 μm pore sieve (Glenammer, Scotland, UK). The samples were further held in a hot-air oven at 121 °C for 1 h to prevent the growth of other microorganisms. The isolation was carried out following the membrane filter technique using Difco^TM^ *Actinomycetes* Isolation Agar (Sparks, MD, USA) [45] with some minor modifications. The cultivated plates were incubated for 4 days at 28 °C until the *Actinomycetes* become visible. The prepared nutrient media was supplemented with 100 μg/mL cycloheximide to suppress eventual growth of fungi. All obtained isolates were cultured in triplicates and further purified for obtaining the pure cultures, which were conserved on slant agar nutrient glycerol (ANG) tubes at 4 °C for further biological assays. The obtained isolates were initially examined based on their microscopic morphological features with a light microscope. For exact identification, the obtained isolates were further analyzed by the molecular method.

### 4.2. Antagonistic Activity

The studied isolates were verified for their biological activity against *E. coli* and *B. megaterium* using the cross-streak method as reported by Odumosu et al. [42]. Briefly, a single, small mass from a fresh culture (24 h) of each studied isolate was streaked in the center of a Petri dish containing KingʹB (KB) nutrient media [46] and then incubated at 37 °C for 48 h. Successively, the plates were inoculated with the tested microorganisms by a single streak at a perpendicular close to the initial inoculum of each studied Actinomycetes isolate. All plates were incubated at 37 °C and the antagonistic activity was evaluated after 24 h. The bacterial antagonistic activity was recorded as follows: very high activity (+++); high activity (++); moderate activity (+); no activity (-). The most bioactive isolates were selected for molecular identification and further in vitro and in vivo biological assays.

### 4.3. Molecular Identification

The bacterial isolates that demonstrated potentially antagonistic effects against the tested target microorganisms were previously morphologically identified under a light microscope (60×) and then by the molecular method based on the analysis of genomic DNA (gDNA) sequences. The gDNA of each studied isolate was extracted using a Qiagen Genomic DNA Kit (Qiagen, Heidelberg, Germany). The extracted gDNA was amplified using the universal primers for bacteria Y1/Y2 (Table 5). The PCR reaction was carried out in a final volume of 25 μL containing: 200 ng DNA, 0.2 µL of 1 U *Taq* DNA polymerase, 2.5 µL *Taq* buffer (20 mM MgCl_2_), 5 µL of each primer (2.5 µM), 5 μL of dNTPs (4 mM) and ultrapure dH_2_O for a final volume of 25 μL. Both the concentration and purity of the total DNA extracted from each sample were measured using a Nano-drop (Thermo Fisher Scientific, Waltham, MA USA). Each DNA sample was subjected to PCR amplification following the cycling profile: 94 °C for 5 min (initial denaturation), followed by 34 cycles of 94 °C for 30 c (denaturation), 57 °C for 30 s (annealing), and 72 °C for 1 min (extension), with a final extension step of 5 min at 72 °C. The amplified DNA, stained by Bromophenol blue (3 µL/10 µL), was applied for agarose gel electrophoresis (1.2%) stained by SYBR green dye (4 µL/100 gel). The obtained amplicons were directly sequenced and compared with those available in the GenBank nucleotide archive using BLAST software [47].

### 4.4. Extracellular Hydrolytic Enzymes

The enzymatic activity of the studied *Actinomycetes* isolates was screened by carrying out an assay of extracellular hydrolytic enzymes on KB media supplemented with the below specific substrates for each enzyme: chitin azure (1%) or chitin from crab shells (1%) for chitinase [48]; skim milk (1%) for protease [48]; and lichenan (0.2%) for glucanase [49]. In addition, soluble starch (1%), pectin (0.5%), and polygalacturonic acid (1%) were used for amylase, pectinase, and polygalacturanase, respectively [50,51]. All plates were incubated at 30 °C for 96 h and then flooded with specific staining solutions as follows: Congo red (0.03%) for chitinase and glucanase; lugol solution for amylase; CTAB: hexadecyltrimethylammonium bromide (2%) for pectinase and ruthenium red (0.1%) for polygalacturanase. The enzymatic activity was taken as evidence of the appearance of hydrolysis clear zones around the colonies, and their diameters were measured in millimeters.

### 4.5. In Vivo Growth Promoting Effect and Disease Control

An in vivo pot experiment was carried out in a greenhouse (School of Agricultural, Forestry, Food and Environmental Sciences-SAFE, University of Basilicata, Potenza, Italy) to evaluate the growth-promoting effect (GPE) of the tested *Actinomycetes* isolates on tomato plants, and the disease control (DC) of the most bioactive isolates was studied against *S. sclerotiorum*.

The pot experiment was carried out in a glass greenhouse at 25 °C for a 15-h photoperiod. Each pot was 20 cm high and 25 cm wide, and previously sterilized with 1.2% sodium hypochlorite for 5 min, rinsed twice with distilled water, and filled with a growing medium mixture (compost/peat moss, 1:1). Seeds of *Solanum lycopersicum* L. cv. *cerasiforme* were surface sterilized by ethanol (70%) and sowed in a cell tray. The temperature and relative humidity in the greenhouse remained stable at 25 ± 2 °C and 70–80%, respectively, for the duration of the experiment.

For the *Actinomycetes* treatment, an initial nutrient culture of peptone yeast calcium agar (PY-Ca) was prepared for the tested isolates and incubated for 5 days at 28 ± 2 °C. A suspension of each studied isolate was prepared by inoculating 10^6^ CFU/mL from the original culture into minimal mineral (MM) media prepared as follows: (g/L) 10.5 K_2_HPO_4_, 4.5 KH_2_PO_4_, 1.0 (NH_4_)2SO_4_, 0.5 Na_3_C_6_H_5_O_7_ × 2H_2_O, 0.2 MgSO_4_ and 5.0 dextrose. The pH value was adjusted at 7.0. The suspensions were then incubated for 7 days at 28 ± 2 °C. The broth cultures were poured into the rhizosphere zone of tomato seedlings (100 mL/pot) 15 days after germination (DAG).

For the fungal artificial infection, Ø 5 mm agar discs from a pure fresh culture (96 h) of *S. sclerotiorum* were inoculated in a sterilized flask filled with potato dextrose broth (PDB) and incubated for 7 days at 22 ± 2 °C. After that, 50 mL of the incubated broth was inoculated in the rhizosphere zone of the seedlings 10 days after the *Actinomycetes* treatment. Ten seedlings were used as the negative health control. The whole experiment was repeated twice with five replicates per treatment. The experimental pots were distributed in a randomized block design in the greenhouse and watered once a day.

For the eco-physiological parameters, plant growth was monitored at the end of the experiment, about 40 DAG, by measuring stem length (SL) in centimeters, number of leaves (NL), number of twigs (NT), the total fresh weights of shoots (TFwS) in grams, and total dry weight of shoots (TDwS) in grams. Regarding the evaluation of the disease incidence, tomato plants were monitored daily, fifteen days after the infection (DAI), to observe the eventual appearance of disease symptoms. The disease incidence was assessed using the following scale (0 = no symptoms observed; 1 = 1 to 20% of leaf chlorosis; 2 = 21 to 50% of leaf chlorosis; 3 = 51 to 80% of leaf chlorosis; 4 ≥ 80% of leaf chlorosis), as reported by Elshafie et al. [4]. The infection percentage (IP %) was measured using Equation (1), whereas the disease index (DI %) and the control effect (CE %) were calculated using Equations (2) and (3), respectively, as described by Lee et al. [52].

IP % = (SL/TL) × 100(1)

DI % = [∑ (Scale × No. of SL)/(HS × TL)] × 100(2)

CE % = 100 × (DI.P−DI.B)/DI.P(3)

where SL is symptomatic leaves; TL is total number of leaves; HS is highest scale; DI-P is disease index of infection; DI-B is disease index of control.

### 4.6. Statistical Analysis

The obtained results were subjected to one-way ANOVA for the statistical analysis. The significance level was checked by applying the Tukey’s B post hoc multiple comparison test with a probability of *p* < 0.05 using Statistical Package for the Social Sciences (SPSS) version 13.0, 2004 (Chicago, IL, USA).

**Table 5:** Primers used in this study.

Primers	Sequences	Target	Amplified Fragment (kb)	Gene	Reference
Y1	5′-TGGCTCAGAACGAACGCTGGCGGC-3′	Bacteria	0.43	16S rDNA	Darrasse et al. [53]
Y2	5′-CCCACTGCTGCCTCCCGTAGGAGT-3′				

## 5. Conclusions

The obtained results of the current research confirmed the promising biological activity of *Actinomycetes*, particularly of the genus *Streptomyces*. This study also underlined the usefulness of the new isolated strains for producing some important bioactive metabolites and extracellular hydrolytic enzymes; hence, they can be effectively used as biocontrol agents against *S. sclerotiorum*. Furthermore, the studied isolates also demonstrated an important plant-growth-promoting effect, which may be due to the production of phytohormones. Further studies remain necessary to identify and biochemically characterize the produced bioactive metabolites from the *Actinomycetes* isolates and evaluate their biological effects against other serious phytopathogens.

## Figures and Tables

**Table 1:** Antagonistic activity of the *Actinomycetes* isolates.

Isolates	Antagonistic Activity	
	*E. coli*	*B. megaterium*
AC1 *	+++	+++
AC2	-	-
AC3	+	+
RS1	+	-
RS2	-	-
RS3 *	+++	+++
FG1	+	++
FG2	+	-
OL1	+	-
OL2 *	+++	++

Note: +++, very high activity; ++, high activity; +, moderate activity; -, no activity. AC1, AC2, and AC3 were isolated from acacia rhizosphere; RS1, RS2, and RS3 were isolated from rosemary rhizosphere; FG1 and FG2 were isolated from strawberry rhizosphere; OL1 and OL2 were isolated from olive rhizosphere. *, isolates that showed the highest antagonistic effect.

**Table 2:** Extracellular hydrolytic enzymes produced by the tested *Actinomycetes* isolates.

Enzyme	Substrates	Staining	Diameter of Hydrolysis Area (mm)		
			AC1 *Streptomyces* sp.	RS3 *S. atratus*	OL2 *A. humicola*
Chitinase	Chitin azure (1%)	Congo red (0.03%)	23.0 ± 2.3 b	0.0 ± 0.0 c	31.5 ± 1.7 a
	Chitin crab shells (1%)	Congo red (0.03%)	0.0 ± 0.0	0.0 ± 0.0	0.0 ± 0.0
Amylase	Soluble starch (1%)	Lugol solution ^(a)^	37.5 ± 2.9 a	42.0 ± 1.2 a	28.0 ± 3.5 b
Glucanase	Lichenan (0.2%)	Congo red (0.03%)	22.0 ± 2.3 b	0.0 ± 0.0 c	36.0 ± 1.2 a
Pectinase	Pectin (0.5%)	CTAB ^(b)^ (2%)	14.0 ± 1.2 a	10.5 ± 1.7 a	0.0 ± 0.00 b
Protease	Skim milk (1%)	-	14.5 ± 2.9 b	12.5 ± 2.9 b	21.5 ± 1.7 a
Polygalacturanase	Polygalacturonic acid (1%)	Ruthenium red (0.1%)	0.0 ± 0.0	0.0 ± 0.0	0.0 ± 0.0

^(a)^ Lugol solution was prepared as follows: 0.35 g iodide + 0.66 g potassium iodide KI in 100 mL dis. H_2_O; ^(b)^ CTAB: hexadecyltrimethylammonium bromide; values followed by different letters in each row for each tested enzyme were significantly different according to *Tukey’s* B multiple comparison test post hoc test at *p* < 0.05.

**Table 3:** Effect of *Actinomycetes* isolates on eco-physiological parameters of tomatoes (health control).

Actinomycetes Isolates	Eco-Physiological Parameters				
	TN (*n*)	SL (cm)	LN (*n*)	SFW (g)	SDW (g)
Cont. -ve	8 ± 1.4 a	36.05 ± 3.2 ab	116 ± 6.9 b	150.02 ± 4.1 b	15.33 ± 1.4 b
AC1: *Streptomyces* sp.	8 ± 0.9 a	39.01 ± 4.1 a	195 ± 11.8 a	204.00 ± 13.4 a	33.02 ± 4.5 a
RS3: *Streptomyces atratus*	6 ± 1.2 a	38.25 ± 7.1 a	123 ± 13.6 b	119.33 ± 8.0 c	15.78 ± 1.9 b
OL2: *Arthrobacter humicola*	7 ± 1.0 a	46.00 ± 3.2 a	151 ± 7.2 a	184.01 ± 7.9 a	24.76 ± 2.7 a

Note: TN: twig number; SL: shoot length; LN: leaf number: SFW and SDW: fresh and dry weight of shoot systems, respectively. Values followed by different letters in each vertical column for each measured parameter were significantly different according to *Tukey’s* B multiple comparison test post hoc test at *p* < 0.05.

**Table 4:** Effect of *Actinomycetes* isolates on eco-physiological parameters of tomatoes (artificially infected with *S. sclerotiorum*).

Actinomycetes Isolates	Eco-Physiological Parameters				
	TW (*n*)	SL (cm)	LN (*n*)	SFW (g)	SDW (g)
Cont. -ve	5 ± 0.2 b	42.32 ± 0.3 a	161 ± 1.0 bc	142.33 ± 1.0 c	24.04 ± 0.2 ab
AC1: *Streptomyces* sp.	8 ± 0.1 a	54.31 ± 0.4 a	333 ± 2.3 a	240.12 ± 2.5 a	30.67 ± 0.3 a
RS3: *Streptomyces atratus*	5 ± 0.1 b	44.34 ± 0.8 a	210 ± 1.8 b	214.67 ± 1.9 ab	23.00 ± 1.0 ab
OL2: *Arthrobacter humicola*	8 ± 0.1 a	51.76 ± 0.2 a	477 ± 3.7 a	304.65 ± 0.8 a	29.67 ± 0.6 a

Note: TN: twig number; SL: shoot length; LN: leaf number: SFW and SDW: fresh and dry weight of shoot systems, respectively. Values followed by different letters in each vertical column for each measured parameter were significantly different according to *Tukey’s* B multiple comparison test post hoc test at *p* < 0.05.

## Data Availability

Not applicable.
